# A Demographic and Regional Comparison of Opioid-Related Hospital Visits within Community Type in the United States

**DOI:** 10.3390/jcm10163460

**Published:** 2021-08-04

**Authors:** Jordan L. Wilkes, Jessica N. Montalban, Brian D. Pringle, Devin Monroe, Adela Miller, Isain Zapata, Amanda E. Brooks, David W. Ross

**Affiliations:** 1 Department of Specialty Medicine, Rocky Vista University, Parker, CO 80134, USA; jordan.wilkes@rvu.edu (J.L.W.); jessica.montalban@rvu.edu (J.N.M.); brian.pringle@rvu.edu (B.D.P.); devin.monroe@rvu.edu (D.M.); adela.miller2920@gmail.com (A.M.); 2 Department of Biomedical Sciences, Rocky Vista University, Parker, CO 80134, USA; izapata@rvu.edu; 3 Office of Research and Scholarly Activity, Rocky Vista University, Ivins, UT 84738, USA; abrooks@rvu.edu

**Keywords:** opioid epidemic, opiate use, regional comparison, Healthcare Cost and Utilization Project, community type

## Abstract

Background: The opioid epidemic is a complex national crisis in the United States with a 400% increase in related deaths over the past two decades with no signs of slowing. The purpose of this study was to assess the incidence of opioid use, based on the geographic and population characteristics. Methods: The opioid-related hospital inpatient stays and emergency department visits obtained from the 2010 to 2018 Healthcare Cost and Utilization Project and demographic confounders, including age, race, education, and income gathered from US Census data were analyzed through generalized linear mixed models and reported by community size and region. Results: Opioid use varies among population center sizes and the region analyzed. In general, opioid visits in the southwest region were greatest across the majority of population center sizes. Rural usage was greatest in the northeast, southeast, and southwest. Unemployment and diverse ethnicities were commonly associated with opioid use in the metro areas studied but these associations were not seen in rural areas. Conclusion: Opioid use remains significant among diverse populations across the United States. Understanding the unique dynamics associated with opioid usage in populations within the regions studied is important in guiding future interventions to fight this crisis.

## 1. Introduction

In the late 1990s, the undertreatment of pain made headlines, leading to the “Decade for Pain Control and Research” in the early 2000s [1]. After this initiative, prescription opioid sales, and subsequent misuse, abuse and addiction, began to rise with opioid-related emergency department (ED) visits and deaths following suit [2]. Opioid use disorders and opioid overdoses have exponentially grown over the past 25 years, mirroring rising levels of opioid prescriptions [3] and increased use of synthetic opioids [4]. Furthermore, current opioid analgesic prescribing practices have been found to lead to increased manifestations of opioid overuse [2,3,5,6,7,8,9,10,11,12].

In the United States, there has been a dramatic rate increase of 64% in opioid-related inpatient hospital stays and a 99% rate increase in ED visits between 2005 and 2014 [7,9,12]. To better stratify these statistics, in 2011, the Drug Abuse Warning Network estimated more than 420,000 ED visits and over 180,000 admissions to treatment centers were related to misuse of opioids [5,8], and from 2009–2014 nearly all states saw a rise in the rate of opioid-related ED visits [9]. This data emphasizes the magnitude of the crisis and its effect on the overall population health of the United States. Separate studies have also shown that many social determinants of health including age, family structure, ethnicity, and gender further impact opioid misuse and overdose rates [13,14,15,16,17].

Opioid use has also exacted a heavy toll on the healthcare system. Published studies from 2010 approximated both indirect and direct costs associated with nonmedical use of prescription opioids to be USD 53.4 billion; abuse, dependence, and misuse USD 55.7 billion; and overdose USD 20.4 billion. Healthcare costs associated with opioids include medical costs related to criminal justice and legal matters, substance abuse treatments, loss of work due to decreased productivity and/or disability, and even premature death [18,19]. In 2009, the estimated cost of ED visits relating to opioid use was USD 800 million and inpatient visits was USD 1.3 billion. More recently published data from 2013 approximates the total economic burden to be USD 78.5 billion [18], while a report in 2015 by the White House Council of Economic Advisers stated the price tag estimation is closer to USD 504 billion [7].

Early studies focused on rural and urban opioid use, suggested increased risk for opioid-related harm in rural communities compared to urban or metropolitan areas [20]. However, more recent analyses reveal rural and urban communities may not be as different as previously thought [6,21]. Newer data indicates opioid-related mortality is more severe in certain rural areas but similar severities can be noted in their metropolitan counterparts [7,22]. The current study extends this analysis to consider not only the rural or urban nature of the setting but also the region of the U.S. and the size of the population center when analyzing opioid usage as indicated by the number of patients presenting to EDs using opioids and number of patients admitted to hospitals using opioids. This study also included an evaluation of social determinants of health demographic factors, including race, ethnicity, unemployment, education, and low socioeconomic status. Specifically, associations between opioid use across communities with diverse population sizes and demographic factors were sought. This study does not evaluate opioid use in general but opioid-related hospital visits. Therefore, the outcome presented is likely to be shifted towards the more severe cases of opioid misuse. An inclusive evaluation of the association of demographic factors to opioid ED and hospital admissions across population centers of diverse sizes can pinpoint areas that will more likely benefit from social programs aimed at reducing the impact of the epidemic.

## 2. Materials and Methods

### 2.1. Opioid-Related Hospital Visit Data

Inpatient and Emergency Department data made available by the Healthcare Cost and Utilization Project (HCUP) was utilized for this study [23]. Data on opioid-related hospital visitation was gathered from the Opioid-Related Hospital Use path, available in the Fast Stats [23]. This path utilized both data from the 2005–2018 State Inpatient Databases (SID) and 2005–2018 State Emergency Department Databases (SEDD). Inpatient statistics from HCUP Fast Stats were available for 47 states and the District of Columbia for various time frames from 2005–2018. The states lacking any inpatient data for this period were Alabama, Idaho, and New Hampshire. HCUP data is not individualized but a compilation of parameter summaries based on a sample that is approximately 20% of all discharges in the United States. ED statistics from HCUP Fast Stats were available for 36 states for various time frames from 2005–2018. The states lacking any ED data for this time frame were Alabama, Alaska, Delaware, Hawaii, Idaho, Louisiana, Michigan, New Hampshire, New Mexico, Oklahoma, Pennsylvania, Virginia, Washington, and West Virginia. Data for the SID included state data from community hospitals, which are short-term, non-Federal government hospitals. These hospitals include obstetrics and gynecology, otolaryngology, orthopedic, cancer, pediatric, public, and academic medical hospitals. Long-term care facilities are excluded from the data; however, patients receiving long-term care in a community hospital were included in the analysis. Similarly, data for the SEDD was limited to state data from community hospitals with a hospital-owned emergency department. The unit of analysis for the inpatient data was hospital discharge by person. Thus, an individual admitted to the hospital multiple times during the course of a single year would be counted each time as a separate statistic. If a patient was admitted to the hospital or transferred to another hospital from the ED, then it was counted in the inpatient data. The unit of analysis for the ED data was the ED visit, not a person or patient, in a similar fashion as inpatient admissions. All patients included in the ED data were discharged from the ED [9].

This study focused on contrasting regional trends between associations of opioid-related hospital visits and demographic factors, with a special focus on communities with different population sizes. Gathered data was organized into several population characteristics within the HCUP database including a total for all populations, age, sex, community-level income, and patient location. The values, which are reported quarterly for each year from 2005–2018, represent rates of either inpatient-related hospital stays or ED discharges per 100,000 US residents. Determination of population size was based on a six-category, county level scheme developed by the National Center for Health Statistics (NCHS), which divided counties into large central metropolitan, large fringe metropolitan (suburbs), medium metropolitan, small metropolitan, micropolitan, and noncore. HCUP combined the micropolitan and noncore categories into one category, deemed rural, creating a total of five patient locations. Community size type descriptions are summarized in Table 1 [24].

### 2.2. Demographic Covariate Data

Confounding covariates other than population size that might affect opioid hospital use were gathered from publicly available state data obtained from the Census and yearly American Community Surveys conducted from 2010 to 2018. The American Community Survey is a stratified sample that is roughly 1% of the total United States population and performed every year outside of the Census years. The data was divided by all 50 states, the District of Columbia, and Puerto Rico for each year. This particular time frame of census data was chosen as it coincided with the data available from HCUP and could be consistently matched to the same time period. Many covariates, such as population density, age, gender, race, education level, and income level, were identified as being important considerations. Within age, the population was divided into categories: under 18, 18–24, 25–65, and over 65. Within the category of race, the population was divided into White (Not Hispanic), Black or African-American, Asian, American/Alaskan Indian, Hispanic or Latino, Pacific Islander, and other. Education was further divided into less than a high school graduate, high school graduate (or its equivalent), some college or associates degree, and bachelor’s degree or more. Finally, income was divided into household adjusted income, percent below the poverty level, and percent unemployed.

### 2.3. Statistical Analysis

Descriptive statistics for census and HCUP data were estimated for the full data set and categorized by the US Department of Health and Human Services defined regions with outcomes being presented as per these defined regions [25]. Inpatient and ED visits by community size type were estimated using HCUP demographic descriptors in combination with census state-wide demographic covariate data. Data was analyzed through generalized linear mixed models (GLMMs) and each of the community size type visits was modeled independently. The dependent variable was square root transformed to correct for a right skewed distribution. Normality assumptions were verified for all models through residual panel fit where all final models displayed no normality assumption violations. Within the model, the US Department of Health and Human Services defined regions were added as a categorical variable while visit type (i.e., inpatient or ED) was considered as a binary categorical variable in the model. All other variables tested in the models were set as continuous with only total population by state values being LN transformed due to scale. To account for the covariance within differences in state reporting, a State categorical variable was introduced to the models as a random effect. Year by year differences were accounted for through the fixed “year” effect.

**Figure 1:**
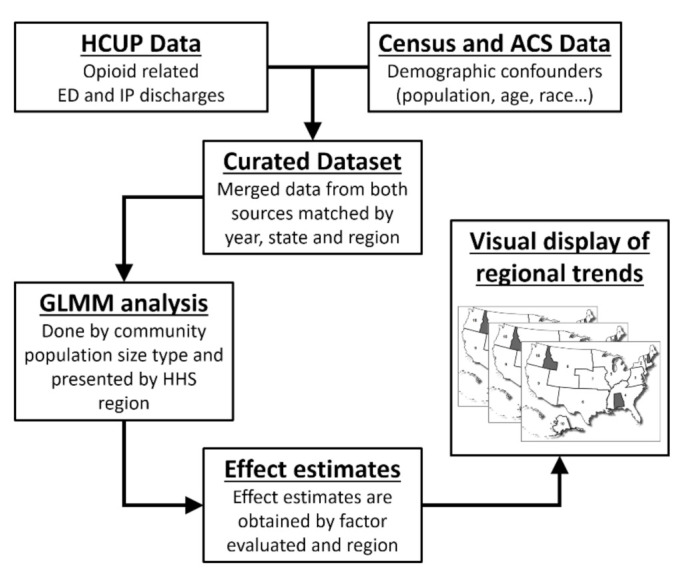
Methodological flow chart of the study. HCUP = Healthcare Cost Utilization Project, ED = emergency department, IP = inpatient, ACS = American Community Survey, GLMM = generalized linear mixed models.

United States Department of Health and Human Services region plots were generated using least square mean estimates for each model fitted. These values were coded with three color gradients (green-white-red) based on their numeric estimate value starting from dark green for the least affected region to dark red for the worst affected region, white being in the center indicating neutrality. HCUP and Census demographic covariate associations were generated and are presented as estimate difference values, where the estimate value represents the value increase per unit of increase or decrease in the covariate units for continuous variables and as a difference from the reference category set arbitrarily for categorical values. All analyses were performed on SAS/STAT v.9.4 (SAS Institute Inc., Cary, NC, USA). Statistical significance was set at *p* ≤ 0.05. Multiple testing correction is highlighted when achieved and was performed through the Bonferroni method. A curated dataset is available as a Supplementary File. A visual representation of the methodological process is displayed in Figure 1.

## 3. Results

The generalized linear mixed model analysis of the data revealed a variety of demographics to be associated with an increased risk of hospital visits. All of the demographics were evaluated and their respective *p*-values are presented in Table 2. Effect estimates for associated covariates are presented in Table 3.

Each population size had a variety of different demographics influencing opioid-related hospital visits. In rural communities, all inpatient stays showed a positive association with opioid visits while hospital settings showed a negative association. In small metro communities, positive associations were seen with all inpatient stays while negative associations were seen with hospital settings, percent under 25, percent White, percent Native American, and percent unemployment. In medium metro communities, positive associations were seen with all inpatient stays, percent White, percent Asian, and percent Native American while negative associations were seen with hospital setting, household adjusted income, and percent unemployment. In large fringe metro communities, positive associations were seen in all inpatient stays, total population, and percent of people aged 25 to 65. In large central metro communities, positive associations were seen in all inpatient stays and percent unemployment while negative associations were seen in sex ratio, percent White, percent Asian, and percent Native American. The percentage of White individuals, percentage of Native Americans, and percent unemployment all appear to have the greatest impact on opioid hospital visits across most population sizes.

**Figure 2:**
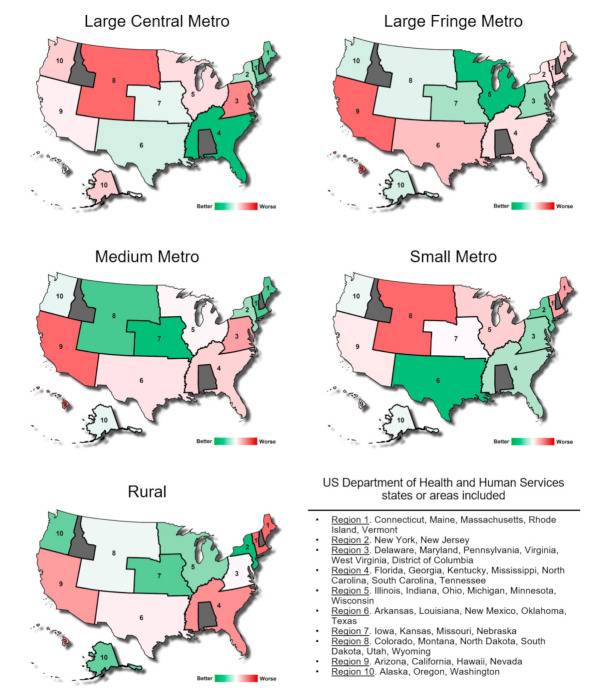
Opioid-related inpatient stays and emergency department visit incidence estimation compared between 10 regions of the United States as defined the Department of Health and Human Services across the five community population sizes. These estimations consider demographic covariates.

The statistical data for inpatient stays and ED visits was combined into one analysis and was thereafter modeled by community population size and United States Department of Health and Human Services region using the geographical areas defined. A visual representation of these regions by community size type is presented in Figure 2. The models generated suggest opioid use varies among population center sizes depending on the region analyzed. Opioid-related visits in Region 9 were greatest across the majority of the community population sizes. In contrast, Region 2 and Region 7 had the least opioid-related visits across the majority of the community population sizes.

Opioid-related visits across metro populations varied greatly from region to region. Large central metro and small metro areas in Region 8 had the highest number of opioid-related visits while large fringe metro and medium metro areas in Region 9 had the highest number of opioid-related visits. Both of these regions include much of the Southwestern and Rocky Mountain regions of the United States. Other large central metro areas with high incidence of opioid-related hospital visits include Regions 3, 5, and 10. Other large fringe metro areas with high incidence included Regions 1, 2, 4, and 6. Other medium metro areas with high incidence included Regions 3, 4, and 6. Other small metro areas with high incidence include Regions 1, 5, and 9.

## 4. Discussion

From 1999 to 2014, the number of opioid-overdose-related deaths in the US almost quadrupled, with more than 400,000 documented mortalities in a 20-year timeframe [5,18,19,26]. This still-growing national crisis has resulted in a 400% increase in deaths associated with opioid use and misuse over the past two decades [7,11,12]. The US Centers for Disease Control and Prevention website highlights relevant opioid research and notes significant increases seen in opioid prescriptions during the 1990s and early years of the new millennium [5]. This phenomenon has also been observed in Canada and European countries where opioid consumption has increased dramatically over the last ten years although the extent of opioid overdoses is not as extensive as in the United States [27,28,29]. These trends have been negatively impacted by the increase in use of synthetic opioids which have fueled the number of fatalities over the last 5 years [4]. These synthetic opioids are often much more powerful than their traditional counterparts [30] and are often obtained from illicit sources linked to the illegal drug trade [31]. These factors increase the risk of overdosing and thus increase the number of emergency hospital visits [32].

The purpose of this study was to assess the community size types and regions of the United States with the highest incidence of opioid-related hospital visits. The assessment was based on the number of patients presenting to EDs secondary to opioids combined with the number patients admitted to hospitals for opioid-related complaints. The prevalence of opioid use is variable across regions of the US and involved a multitude of demographic factors [22]. For example, the data revealed that rural areas are inconsistent when it comes to the incidence of opioid-related inpatient and ED stays. For rural communities, regions in the west of the United States appear to be most affected by the opioid crisis across all population sizes as previously reported [22], while communities in the northwest coastal region appear to be the least affected. Notably, in the northwest region, the impact of the opioid epidemic seems to worsen when moving from rural population densities to more urban population densities. Although, the economic situation of the region plays an important role for opioid abuse victims [32,33] we observe in our study that population size centers in any other regions are mixed with no clear trends, supporting the idea that no single entity is responsible for the cause or the complexity of this issue.

The percentages of White, Native American, and unemployed people have the strongest association to opioid-related hospital visits. However, the association with population size was variable. Previous studies between 1999 and 2016 found the “age-adjusted opioid-related mortality increased by 158% in Large Central Metro counties, 507% in Large Fringe Metro counties, 388% in Medium Metro counties, 584% in Small Metro counties, 682% in Micropolitan Non-Metro counties, and 721% in Noncore Non-Metro counties” [7]. Similarly a study evaluating synthetic opioid-related fatalities between 2013 and 2019 showed a large spike in illegal synthetic drug use which is replacing a declining over-prescription trend [4]. An explanation as to why unemployed and population size variables have a higher risk of opioid-related hospital visits can be very complex and cannot be deduced from our data. However, it has been proposed that certain adverse events can lead to social isolation, increasing the risk of opioid abuse as a coping mechanism [34]. Conversely, the current study did not find age to be a significant factor, and there are no other reports with comparable demographic substratifications to provide further context. While the Rigg et al. study utilized population size areas similar to the current study, it did not further stratify data into regions, though it did note regional differences between the broadly defined Northeast, Midwest, Southern, and Western regions. It also reported details as to how these particular trends obscured important regional differences. Furthermore, it showed higher rates of opioid use since the late 2000s as previously reported in large central metro population centers in the northeast area of the US [7,35].

Alternatively, the current study used a novel analysis which found several trends. It was found that higher opioid usage correlates with Region 1. The Midwest had the highest opioid use, which correlates with Regions 6, 7, and 8 in this study. Usage was also consistently high in large central metro population centers in this study. The current study used additional regional stratifications, allowing high opioid usage levels to be identified in large central metro areas in Region 8, in small metro areas in Region 8, and in large fringe metro areas in Region 6, but not in medium metro or rural areas. Previous studies also found rates of opioid usage were highest in the large fringe metro and medium metro areas in the southern region. This trend held true in the current study and was particularly evident in Region 6; however, the current study also found rural areas in this region not lagging far behind in their opioid usage. In addition, Region 4 showed high rates of opioid usage in rural areas but not in small metro or large central metro population centers. Further to the West, past studies (i.e., since the mid-2000s) found rates to be consistently higher in small and medium metro areas and non-metro areas. Correlating the data from these previous studies with the analogous Regions 9 and 10 in the current study, it becomes essentially impossible to determine a definitive trend in opioid usage among different size population centers. While Region 9 has the highest incidence of opioid-related hospital visits in medium metro and large fringe metro areas followed closely by rural areas, Region 10 shows comparably lower incident levels with the notable exception of large central metro areas.

The analysis performed as part of our study shows the impact of opioid-related hospital visits is intimately related to specific demographic descriptors and tends to vary across geographical regions, giving a more complete picture of the opioid crisis across the entire US. Furthermore, since the current study utilized the HSS regional breakdown, it can be directly used by those creating regional policy to further inspect and analyze the causes of increased visits due to opioid usage in particular regions as well as population size areas. Moreover, this analysis can be employed in conjunction with public health measures to provide support to stem the opioid epidemic locally. Although there is some understanding of the factors fueling the opioid crisis, this paper provides an important analytical tool to better identify key regional contributors and covariables to the opioid epidemic. These regional differences also suggest a single generalized nationwide approach to mitigate the impact of the opioid epidemic is not likely to be effective. Armed with this knowledge, targeted interventions may be identified to reduce opioid misuse and its associated negative effects.

The use of a large, multiyear, hospital database coupled with employment data from the census allowed the assessment of general trends of opioid use. The Census in particular “can be an essential tool for understanding county-level variation in drug mortality rates and in driving policy responses to the crisis” [11]. The current study provides important new information for more localized regions of the nation, which can be applied to specific communities based on their size. The current study identified information for different population sizes as well as certain demographics of the populations that are at the highest risk for opioid-related hospital visits.

Despite the important findings outlined in this report, it is not without its limitations. Over the past few years, the opioid crisis has continued to evolve, developing into an opioid epidemic [36,37]. Subsequently, the data related to the opioid epidemic has also changed [38,39,40]. This is a result of differences in prescribing practices and the overall attitude and approach toward opioid prescribing. Opioid prescription in the United States is regulated by federal and state guidelines that when combined can lead to discrepancies in prescription practices across states. The burden of over prescription has decreased over the years but has been replaced by the increase of illicit use of synthetic opioids distributed through the illegal drug trade [4,31]. This study attempted to utilize the most complete data that remained relevant to the time period that was studied (2010–2018). For instance, the Center for Disease Control (CDC) has released more current data since 2018. However, not all aspects of the data that was analyzed in this study was available from the CDC data. Therefore, an older, more complete data set was utilized to offer a more accurate picture of the opioid epidemic during a relevant time period.

Since this study measured hospital visits, not the use of opioids in general, the data may be biased towards the more severe cases of opioid misuse, ultimately falling short of addressing the opioid burden as a whole. Furthermore, the current data was limited by the reporting date ranges since some states only provided a few years of data while other states have no data reported to HCUP. Another limitation was the use of ICD-9-CM and ICD-10-CM codes from records of patients who were admitted to the hospital or discharged from the ED to determine opioid-related hospital stays. ICD-10-CM were utilized for all data after 1 October 2015 when these codes replaced ICD-9-CM codes [41]. ICD 9 and 10 coding are dependent on both the charting of the individual provider and their choice of the appropriate code. Notably, there is an element of subjectivity in both the charting and coding, which could affect the data obtained from HCUP [23]. Future research should be directed at measuring the impact that changes in coding have on reporting and measuring opioid-related hospital visits.

## 5. Conclusions

The current study demonstrated how population centers across various regions are affected differently by the opioid epidemic. The factors impacting population centers of similar sizes can be different from region to region; these differences must be considered to develop more effective strategies to mitigate opioid misuse. Health professionals and public health officials must understand and consider the specific conditions of their locality to better serve their community [42]. The data in this study indicates opiate use remains significant among diverse populations across the United States. Understanding the unique dynamics associated with opioid-related hospital visits in populations within the regions studied is important for guiding future interventions to fight this crisis. Further research should explore the causes of the opioid use variability in each population density within each region.

## Figures and Tables

**Table 1:** Summary of community population sizes evaluated in this study.

Community Size Type	Description
Large central netro	Counties in metropolitan statistical areas (MSAs) of 1 million or more population that contain the entire population of the largest principal city of the MSA, have their entire population contained in the largest principal city of the MSA, or contain at least 250,000 inhabitants of any principal city of the MSA.
Large fringe metro	Counties in MSAs of 1 million or more population that did not qualify as large central metropolitan counties.
Medium metro	Counties in MSAs of populations of 250,000 to 999,999 inhabitants.
Small metro	Counties in MSAs of population less than 250,000 inhabitants.
Rural	Counties in micropolitan statistical areas and other nonmetropolitan counties.

**Table 2:** Demographic covariate association to opioid-related hospital visits compared across different community population sizes in the entire United States. * Significant at *p* ≤ 0.05. ** Significant at Bonferroni adjusted *p*.

Effect		Rural	Small Metro	Medium Metro	Large Fringe Metro	Large Central Metro
	Year	0.0173 *	0.6158	0.0978	0.0662	0.2024
	Region	0.0068 *	0.0526	0.7631	0.0440 *	0.0220 *
	Hospital Setting	5.6E-10 **	3.5E-18 **	0.0230 *	0.6325	0.2670
	All Inpatient Stays	8.3E-63 **	1.1E-27 **	1.3E-83 **	1.1E-83 **	3.1E-84 **
**Population**	Total population (LN)	0.9903	0.0742	0.3347	0.0087 *	0.5864
	Population Density	0.3039	0.5301	0.1120	0.3751	0.2355
	Sex Ratio	0.7881	0.6347	0.2171	0.6009	0.0072 *
**Age**	% Under 25	0.3341	0.0391 *	0.3676	0.1979	0.5547
	% 25 to 65	0.9127	0.3929	0.3521	0.0462 *	0.3990
	% 65 and over	0.4891	0.1442	0.2020	1.0000	1.0000
**Race**	% White Alone	0.7485	0.0239 *	0.0495 *	0.4587	0.0447 *
	% African-American	0.9024	0.0681	0.0711	0.7270	0.0694
	% Asian	0.3011	0.1842	0.0080 *	0.8004	0.0348 *
	% Native American	0.8485	0.0038 *	0.0052 *	0.8062	0.0311 *
	% Hispanic	0.9594	0.0603	0.0607	0.8827	0.0561
**Education**	% No High School	0.8248	0.5890	0.2024	0.2893	0.7425
	% High School	0.4069	0.7209	0.2026	0.5208	0.7715
	% Some College	0.2828	0.6936	0.1957	0.2164	0.7529
	% Bachelors or more	0.4021	0.9609	0.5795	0.7986	0.5975
**Income**	Household Adjusted Income	0.9076	0.4611	0.0033 *	0.9670	0.9979
	% Poverty Level	0.3760	0.1402	0.7839	0.6598	0.7576
	% Unemployment	0.4837	0.0005 *	0.0120 *	0.3315	3.5E-5 **

**Table 3:** A list of demographic variables within each population size for the entire United States that were found to be statistically significant. Values are square root transformed. ED = emergency department discharge. IP = inpatient discharge.

Effect by Population Type	Estimate	Standard Error	95% Confidence Limits
**Rural**			
Hospital Setting ED vs ID	−0.7219	0.114	(−0.946, −0.498)
All Inpatient Stays	0.021	0.001	(0.019, 0.024)
**Small Metro**			
Hospital Setting ED vs ID	−1.4378	0.159	(−1.750, −1.125)
All Inpatient Stays	0.018	0.002	(0.015, 0.021)
% Under 25	−0.9129	0.442	(−1.780, −0.046)
% White Alone	−0.7876	0.348	(−1.471, −0.105)
% Native American	−1.2863	0.443	(−2.157, −0.416)
% Unemployment	−0.3691	0.105	(−0.575, −0.163)
**Medium Metro**			
Hospital Setting ED vs. ID	−0.2493	0.109	(−0.464, −0.034)
All Inpatient Stays	0.026	0.001	(0.024, 0.028)
% White Alone	0.548	0.278	(0.001, 1.094)
% Asian	1.021	0.384	(0.267, 1.775)
% Native American	1.054	0.376	(0.316, 1.792)
Household Adjusted Income	−0.00012	0.00004	(−0.00021, −0.00004)
% Unemployment	−0.1874	0.074	(−0.334, −0.041)
**Large Fringe Metro**			
All Inpatient Stays	0.027	0.001	(0.025, 0.029)
Total population (LN)	2.131	0.809	(0.541, 3.722)
% 25 to 65	0.548	0.274	(0.009, 1.086)
**Large Central Metro**			
All Inpatient Stays	0.039	0.002	(0.036, 0.042)
Sex Ratio	−82.7949	30.670	(−143.090, −22.496)
% White Alone	−0.953	0.473	(−1.883, −0.023)
% Asian	−1.3688	0.646	(−2.639, −0.098)
% Native American	−1.6092	0.744	(−3.071, −0.147)
% Unemployment	0.485	0.116	(0.257, 0.712)

## Data Availability

All data used in this study is available in the public domain. HCUP data can be accessed at https://www.hcup-us.ahrq.gov/ (accessed on 23 July 2020) while demographic covariate data can be accessed at https://data.census.gov/cedsci/ (accessed on 29 July 2020). A curated dataset is available as a Supplementary File.

## Supplementary Materials

The following are available online at https://www.mdpi.com/article/10.3390/jcm10163460/s1, Table S1: Curated Dataset.
